# 

**Published:** 1855-07

**Authors:** 


Messrs. A. I. Mathews & Co., of this city, have issued their Annual Catalogue, a beautiful pamphlet of 160 pages, in which physicians are informed at what prices they may procure reliable drugs, surgical instruments, etc. Those who send orders to this house may rely on receiving good articles at fair prices.

*New Books.*—Gross on Diseases of the Urinary Organs, Ashwell on Diseases of Females, and Tyler Smith on Leucorrhœa, are received from the publishers, and shall have early notice.

---

*Business Matters.*—We inclose receipts, in this number, for money received, to a large number of our subscribers. To those of them—and they are not a few—who have been so kind as to send a word of approval and encouragement with the money, we offer our thanks. Nothing can be more grateful to us than such expressions, and nothing is a better stimulus to labor.

Our anticipations as to new subscribers have been fully realized, our edition of the June number being almost exhausted. We have it in contemplation, should a sufficient number of new subscriptions be received at an early day, to issue a second edition of that number, so that our readers may possess the full volume.

In the meantime we must claim the right to urge upon those still indebted to us for former subscriptions, to forward their dues without delay.

Mr. J. Clement, just about starting on a western tour, will present our accounts to subscribers in Michigan and Wisconsin, and is authorized to sign receipts. Our friends in these states, however, might serve as patterns for those nearer home, as no part of our mail list shows so prompt a class of subscribers as those in Michigan and Wisconsin.

A Case of Placenta Prævia, from our good friend, Dr. Hershey, of Freeport, Ill., is received, and will find room in our next. We call the attention of our readers to the neatness and accuracy of the lithograph, by Compton, accompanying the present number. Now that we have ascertained that good anatomical plates can be executed in Buffalo, we shall make use of them whenever they will assist the text.

